# Development of a multiplex qRT-PCR assay for detection of classical swine fever virus, African swine fever virus, and *Erysipelothrix rhusiopathiae*

**DOI:** 10.3389/fvets.2023.1183360

**Published:** 2023-05-25

**Authors:** Liang Zhao, Xiao-Hui Wen, Chun-Ling Jia, Xiu-Rong Zhou, Sheng-Jun Luo, Dian-Hong Lv, Qi Zhai

**Affiliations:** ^1^Key Laboratory of Livestock Disease Prevention of Guangdong Province, Scientific Observation and Experiment Station of Veterinary Drugs and Diagnostic Techniques of Guangdong Province, Ministry of Agriculture and Rural Affairs, Institute of Animal Health, Guangdong Academy of Agricultural Sciences, Guangzhou, China; ^2^College of Animal Science, Tibet Agriculture and Animal Husbandry University, Linzhi, China

**Keywords:** classical swine fever virus (CSFV), African swine fever virus (ASFV), *Erysipelothrix rhusiopathiae*, multiplex qRT-PCR, detection

## Abstract

Classical swine fever virus (CSFV), African swine fever virus (ASFV), and *Erysipelothrix rhusiopathiae* (*E. rhusiopathiae*) remain endemic in many parts of China. Co-infections make distinguishing their clinical symptoms and pathological changes difficult. This study developed a multiplex real-time quantitative reverse transcription polymerase chain reaction (multiplex qRT-PCR) that can simultaneously detect CSFV, ASFV, and *E. rhusiopathiae*. Three sets of primers and probes were designed to target the CSFV 5΄ untranslated region, ASFV p72 gene, and *E. rhusiopathiae* 16sRNA gene. Multiplex qRT-PCR for simultaneous differential detection of these three pathogens was developed after optimizing reaction parameters such as annealing temperature, primer and probe concentrations, amplification cycles, etc. The multiplex qRT–PCR could detect CSFV, ASFV, and *E. rhusiopathiae* simultaneously but could not amplify other porcine pathogens. The assay’s limit of detection (LOD) was 2.89 × 10^2^ copies/μL for CSFV, ASFV, and *E. rhusiopathiae*. All correlation coefficients (R^2^) at higher than 0.99, and the amplification efficiency was 98, 90, and 84%, respectively. All correlation coefficients (R2) were higher than 0.99, and the efficacy of amplification was 84%. In a repeatability test utilizing standard recombinant plasmids, the intra- and inter-assay coefficients of variation (CVs) were less than 2.27 and 3.79 percent, respectively. Lastly, 150 clinical samples were used to evaluate the assay’s applicability in the field. The positive rates of CSFV, ASFV, and *E. rhusiopathiae* were 1.33%, 0, and 3.33%, respectively. And no co-infection among the three pathogens was found. The concordance rate between the multiplex qRT-PCR and single-plex commercial PCR kits reached 100%. This study’s multiplex qRT-PCR could provide a rapid, sensitive, and specific method for the simultaneous and differential detection of CSFV, ASFV, and *E. rhusiopathiae*.

## 1. Introduction

Classical swine fever virus (CSFV), a single-stranded, positive-sense RNA virus in the *Pestivirus* genus of the *Flaviviridae* family (1), is the causative agent of classical swine fever (CSF), which frequently manifests as fever, organ hemorrhage, and immunosuppression (2). The African swine fever virus (ASFV), an enveloped double-stranded DNA virus, is currently the sole member of the *Asfivirus* genus within the *Asfarviridae* family (3). This virus can cause African swine fever (ASF), typified by high fever, pulmonary edema, severe hemorrhage, and extensive necrosis of lymphoid tissue, with close to 100% higher morbidity and mortality (4). *Erysipelothrix rhusiopathiae* (*E. rhusiopathiae*), a nonsporulating, gram-positive, rod-shaped bacterium, pertains to the *Erysipelothrix* genus. *E. rhusiopathiae* can cause swine erysipelas, displaying the primary clinical manifestation with fever, lameness, diamond-shaped lesions on the skin, and sudden mortality in growing and adult swine (5, 6). CSFV, ASFV, and *E. rhusiopathiae* are still prevalent in many countries, resulting in significant economic losses for the global swine industry. Similar clinical signs and pathological alterations make it difficult to distinguish between CSF, ASF, and swine erysipelas in the field. To diagnose these diseases accurately and quickly, developing a specific, sensitive, and rapid test method to detect these pathogens simultaneously and discriminately is essential.

Multiplex real-time quantitative polymerase chain reaction (multiplex qRT-PCR) offers excellent performance in clinical testing, which is based on constant measurements of the change of fluorescent signals during the amplification reaction (7). It provides greater detection capacity, faster speed, and lower labor costs (8). Most multiplex qRT-PCR assays are based on target-specific TaqMan probes, which have higher specificity than conventional RT-PCR, and it is widely used for pathogen diagnosis and monitoring (9). Currently, several qRT-PCR and qPCR assays have been established for the detection of CSFV (10, 11), ASFV (12, 13), *E. rhusiopathiae* (14, 15), and CSFV+ASFV (16, 17). However, a multiplex qRT–PCR assay that can simultaneously and differentially detect CSFV, ASFV, and *E. rhusiopathiae* has not been reported. Hence, developing a rapid and specific method for differentiating between these three pathogens is essential. This study aimed to establish multiplex qRT-PCR method-based TaqMan probes for simultaneously and differentially detecting CSFV, ASFV, and *E. rhusiopathiae*.

## 2. Materials and methods

### 2.1. Viral nucleic acids, viruses, bacteria, and clinical samples

Eight ASFV-positive nucleic acid samples were provided by the Guangdong Animal Disease Prevention and Control Center. Type O foot-and-mouth disease virus (FMDV-O) nucleic acids, type A foot-and-mouth disease virus (FMDV-A) nucleic acids, CSFV nucleic acids, *E. rhusiopathiae*, porcine epidemic diarrhea virus (PEDV), porcine delta coronavirus (PDCoV), transmissible gastroenteritis virus (TGEV), emerging swine acute diarrhea syndrome coronavirus (SADS-CoV), porcine reproductive and respiratory syndrome virus (PRRSV), porcine rotavirus (PRV), porcine circovirus 2 (PCV2), swine *Escherichia coli*, and swine *Pasteurella multocida* were preserved by the Institute of Animal Health of the Guangdong Academy of Agricultural Sciences. A total of 150 clinical samples were obtained from pig farms in the southern Chinese province of Guangdong between 2021 and 2022, including 107 blood samples, 35 lymph node samples, and 8 kidney samples.

### 2.2. Design of primers and probes

The study involved the design of specific primers and corresponding TaqMan probes targeted toward the 5′ untranslated region (UTR) of CSFV, the p72 gene of ASFV, and the 16sRNA gene of *E. rhusiopathiae*. Then the primers and probes were synthesized by Sangon Biotech Co. Ltd. (Shanghai, China). Table 1 lists detailed data relating to the primers and probes.

**Table 1:** Primers and probes for the detection of CSFV, ASFV, and *Erysipelothrix rhusiopathiae.*

Pathogens	Names	Sequences (5′→3′)	Product size (bp)
CSFV	CSFV-F	GTGGTCTAAGTCCTGAGTA	107
	CSFV-R	GGGTTAAGGTGTGTCTTG	
	CSFV-Probe	FAM-CCTCGTCCACRTAGCATCTCG-BHQ1	
ASFV	ASFV-F	AGACGCATGTTCATCTATA	127
	ASFV-R	GCAGAACTTTGATGGAAA	
	ASFV-Probe	VIC-TCCGTAACTGCTCATGGTATCAATCT-BHQ1	
*E. rhusiopathiae*	ER-F	GCCATAGAAACTGGTAGA	217
	ER-R	GCACTGAATTTCTCCAAC	
	ER-Probe	ROX-CTAGTAGTCCACGCCGTAAACGA-BHQ2	

### 2.3. Nucleic acid extraction

Major swine pathogens and clinical samples nucleic acids were extracted using GeneRotex96 automatic nucleic acid extractor with Tianlong virus DNA / RNA extraction kit and bacterial genomic DNA extraction kit (Xi’an TianLong Science and Technology Co., Ltd., Xi’an, China) according to the manufacturer’s instructions and stored at −80°C until use.

### 2.4. Construction of standard plasmids

Recombinant plasmid pUC57-CSFV-ASFV-ER contained the 267 bp gene sequence, including three target amplified fragments was synthesized by Tsingke Biotechnology Co., Ltd. DNA concentration was determined by Thermo Scientific NanoDrop Lite (Wilmington, DE, United States). And the copy number was calculated according to the following equation:

$$Numberofcopies\;per\;\mu L=\frac{c\times 6.022\times 10^{23}}{n\times 660}$$

where c = concentration of pUC57-CSFV-ASFV-ER (g/μL); n = number of base pairs in a single pUC57-CSFV-ASFV-ER.

### 2.5. Optimization of the reaction conditions of the multiplex qRT-PCR

Optimizing multiplex qRT-PCR conditions, encompassing annealing temperature, primer and probe concentrations, amplification cycles, and other relevant parameters, was conducted using Analytik Jena qTOWER3/G (Jena, Germany). The optimal reaction conditions for the multiplex qRT-PCR were determined using the following 20 μL basic systems:10 μL of One Step PrimeScript III RT-qPCR (2×) (Takara, Dalian, China), 2.0 μL of standard plasmid (containing 2.89 × 104 copies/μL), and three pairs of primers and probes with varying final concentrations, along with distilled water. The amplification parameters were as follows: reverse transcription at 52°C for 5 min, predenaturation at 95°C for 10 s, 40 cycles of 95°C for 5 s, varying annealing temperature for 30 s, and scanning for the fluorescence signal at the final stage in each process. The following reaction conditions parameters were obtained through an arrangement and combination test: the annealing temperature was from 53.1 to 59.9°C; the concentrations of the probe were 50, 75, 100, and 125 nM, respectively; the concentrations of the primers were100, 150, 200, 250 nM, respectively; amplification cycles were 35, 40, 45, respectively. Optimizing the final concentrations of the primers, probes, and amplification conditions were conducted to achieve the highest fluorescence intensity and the lowest threshold cycle (CT) by utilizing the standards plasmid of 2.89 × 104 copies/μL as templates. The specificity assay, sensitivity assay, reproducibility assay, and detection of clinical samples in the following study were conducted under the optimized reaction conditions.

### 2.6. Analytical specificity assay

The specificity of the established multiplex qRT-PCR was estimated by utilizing standard DNAs or RNAs of prominent swine pathogens, such as CSFV, ASFV, *E. rhusiopathiae*, PEDV, PDCoV, TGEV, SADS-CoV, FMDV-O, FMDV-A, PRRSV, PRV, PCV2, swine *Escherichia coli*, and swine *Pasteurella multocida* as templates for amplification. Nuclease-free water was utilized as a negative control, while standard plasmids were employed as a positive control.

### 2.7. Analytical sensitivity assay and standard curves

To determine the assay’s sensitivity, the pUC57-CSFV-ASFV-ER was serially diluted 10-fold 10 times with concentrations ranging from 2.89 × 10^−1^ to 2.89 × 10^8^ copies/μL. The detection limit was verified by amplifying diluted standard plasmids using the optimized qRT-PCR system. The optimized RT-qPCR assay ran the pUC57-CSFV-ASFV-ER dilutions of 2.89 × 10^2^ to 2.89 × 10^7^ copies/μL and established three standard curves calculated and plotted automatically by qPCRsoft 4.0.

### 2.8. Repeatability assay

The repeatability assay included intra- and inter-assay measurements. The DNA templates used in the repeatability test were diluted to 2.89 × 10^3^, 2.89 × 10^4^, and 2.89 × 10^5^ copies/μL of pUC57-CSFV-ASFV-ER. Intra-assay was conducted by performing one repeat of the assay on each of the three dilutions, with nine replicates for each dilution. Inter-assays were conducted by performing the assay three times on each of the three dilutions on three separate occasions.

### 2.9. Detection of clinical samples

A total of 150 clinical samples were extracted and tested with the optimized multiplex real-time PCR assay. Positive (RT-qPCR system with pUC57-CSFV-ASFV-ER plasmid as template) and negative (RT-qPCR system with nuclease-free water as template) controls were included in each run.

### 2.10. Comparison of multiplex qRT-PCR with single-plex commercial PCR kits

Two CSFV-positive and five *E. rhusiopathiae*-positive nucleic acids from the clinical samples, along with eight ASFV-positive nucleic acids, were confirmed through Sanger sequencing. These nucleic acids, including 20 negative clinical samples, were subsequently amplified using both the established multiplex qRT-PCR assay and the single-plex commercial PCR kits. The ASFV commercial PCR kit was produced by Beijing SCENK biotechnology development Co., Ltd. (Beijing, China), while the CSFV and *E. rhusiopathiae* commercial PCR kits were produced by Aodong Inspection & Testing Co., Ltd. (Shenzhen, China). The diagnostic sensitivity and specificity of the assay were evaluated using commercial PCR kits as the standard.

## 3. Results

### 3.1. Optimal reaction conditions of the multiplex qRT-PCR

**Figure 1:**
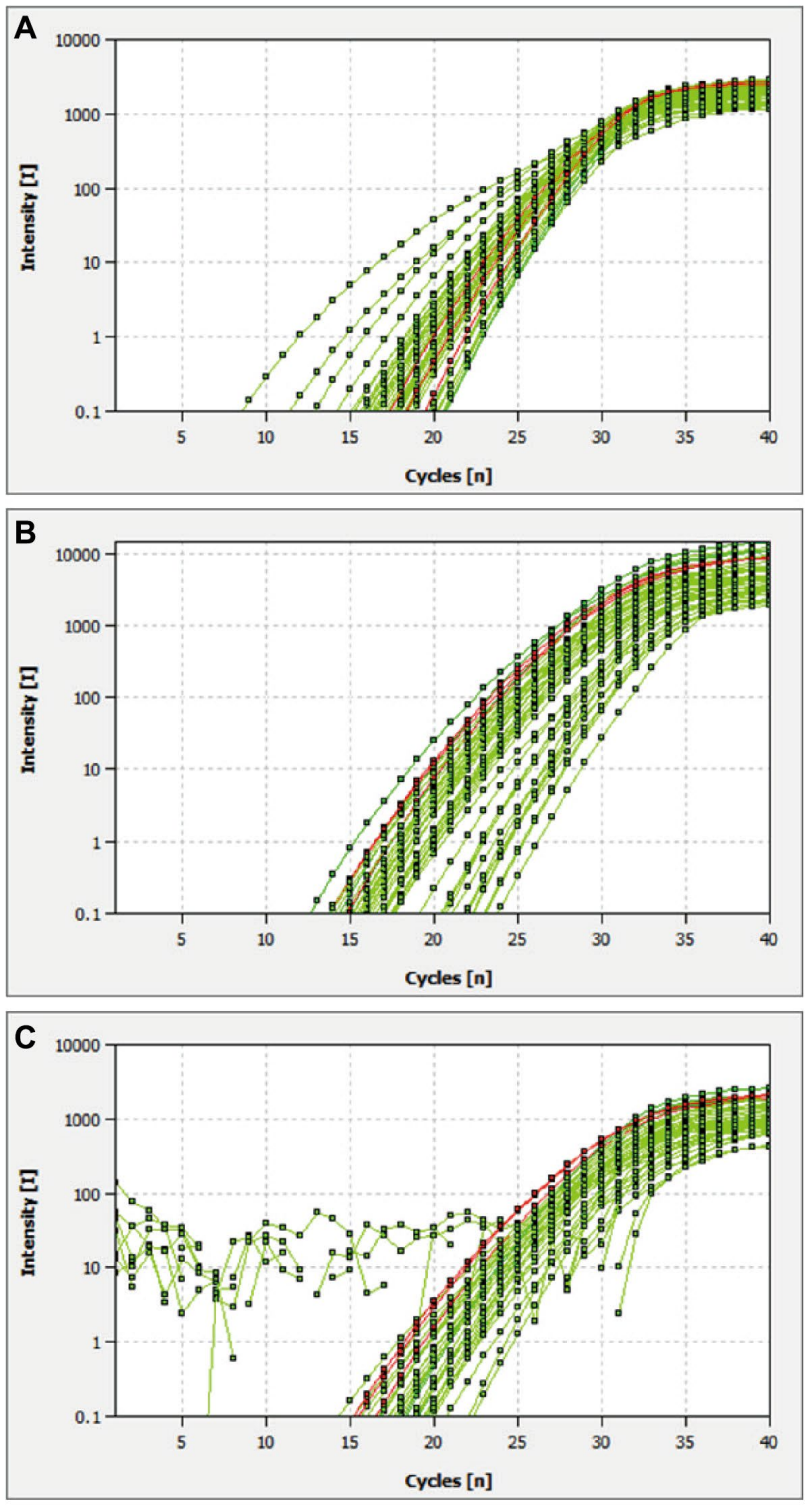
Optimal reaction conditions of the multiplex qRT-PCR. **(A–C)** Amplification curves of CSFV, ASFV, and *E. rhusiopathiae* detected by multiplex real-time PCR with different probe and primer concentrations. Plasmid standards with a concentration of 2.89 × 10^4^ copies/μL were chosen as reaction templates. The red lines are the amplification curves of three fluorescence of the most suitable reaction tube.

The multiplex qRT-PCR was performed using probes concentrations ranging from 50 to 125 nM and primers with concentrations ranging from 100 to 250 nM. The fluorescence intensity and Ct values of all possible combinations were compared. After optimization, the final concentrations for probes and primers were determined to be 125 and 250 nM, respectively (Table 2; Figure 1). The developed multiplex qRT-PCR with a total volume of 20 μL, containing 10 μL 2× One-Step PrimerScript III RT-PCR (TaKaRa), 0.50 μL forward primer (10 μM), 0.50 μL reverse primer (10 μM), 0.25 μL probe (10 μM), 4.25 μL RNase-free ddH_2_O, and 2.0 μL template. The amplification process was conducted with the following parameters: reverse transcription at 52°C for 5 min, predenaturation at 95°C for 10 s, followed by 45 cycles of denaturation at 95°C for 5 s, and annealing and extension at 59°C for 30 s. Fluorescent signals were collected at the end of each cycle.

**Table 2:** This multiplex real-time PCR assay detected mean Ct values of CSFV, ASFV, and ER with different probe and primer concentrations.

CFSV (*n* = 3)				
Probe concentration (nM)	Primer concentration (nM)			
	100	150	200	250
50	31.21 ± 0.91	30.68 ± 0.54	30.23 ± 0.40	30.38 ± 0.28
75	29.65 ± 1.09	29.88 ± 0.27	29.65 ± 0.21	29.82 ± 0.63
100	29.72 ± 0.51	28.99 ± 0.13	29.58 ± 0.29	28.94 ± 0.20
125	29.28 ± 0.32	29.91 ± 0.48	30.55 ± 0.19	29.54 ± 0.22
ASFV (*n* = 3)				
Probe concentration (nM)	Primer concentration (nM)			
	100	150	200	250
50	34.25 ± 0.33	32.19 ± 0.62	31.33 ± 0.85	30.79 ± 0.50
75	34.23 ± 0.77	31.86 ± 0.98	29.34 ± 0.25	29.51 ± 0.39
100	32.68 ± 0.68	30.17 ± 0.56	29.01 ± 0.30	29.10 ± 0.09
125	30.13 ± 0.34	28.63 ± 0.48	27.40 ± 0.44	27.96 ± 0.26
ER (*n* = 3)				
Probe concentration (nM)	Primer concentration (nM)			
	100	150	200	250
50	NC	NC	NC	30.02 ± 0.63
75	NC	30.98 ± 1.00	30.09 ± 0.16	29.22 ± 0.23
100	NC	29.69 ± 0.51	29.41 ± 0.16	28.69 ± 0.58
125	30.12 ± 0.67	28.77 ± 0.28	28.45 ± 0.41	27.47 ± 0.41

### 3.2. Sensitivity and standard curves of the multiplex qRT-PCR

**Figure 2:**
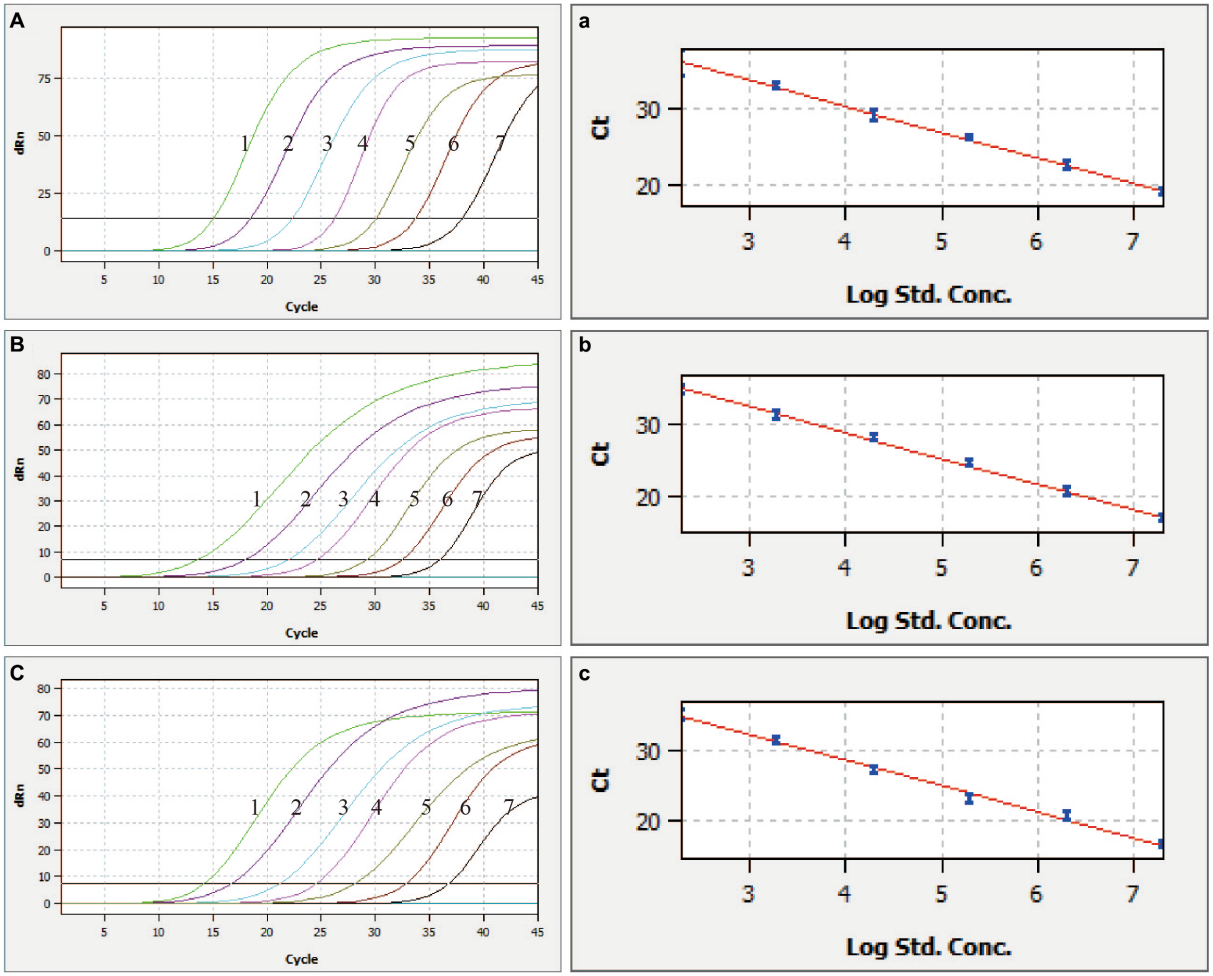
Sensitivity and standard curves of the multiplex qRT-PCR. **(A–C)** Fluorescence curve of CSFV, ASFV, and *E. rhusiopathiae*, **(a–c)** the standard curve of CSFV, ASFV, and *E. rhusiopathiae*, 1–7: 2.89 × 10^8^ copies/μL – 2.89 × 10^2^ copies/μL of the standard plasmids.

The multiplex qRT-PCR demonstrated high sensitivity, as evidenced by the valid positive result obtained at the lowest copy number of 2.89 × 10^2^ copies/μL (Figure 2). Multiplex qRT-PCR standard curves were constructed by 10-fold serial dilution of plasmids ranging from 2.89 × 10^7^ copies/μL to 2.89 × 10^−1^ copies/μL as templates. The correlation coefficient (R^2^) and amplification efficiency (Eff%) of the equation were as follows: CSFV (*R*^2^ = 0.999, Eff% = 98); ASFV (*R*^2^ = 0.999, Eff% = 90); *E. rhusiopathiae* (*R*^2^ = 0.996, Eff% = 84), indicating a strong linear correlation between the initial template and the Ct value (Figure 2).

### 3.3. Specificity of the multiplex qRT-PCR

**Figure 3:**
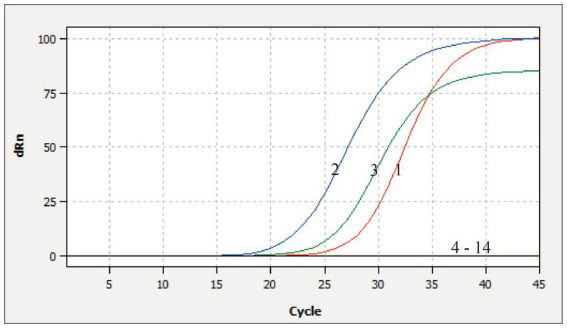
Specificity analysis of this multiplex qRT-PCR. 1–3: Positive templates of CSFV, ASFV, and *E. rhusiopathiae*. 4–14: PEDV, PDCoV, TGEV, SADS-CoV, FMDV-O, FMDV-A, PRRSV, PRV, PCV2, swine *Escherichia coli*, swine *Pasteurella*, and nuclease-free water control.

The DNA or RNA from various porcine pathogens were used as templates for the multiplex qRT-PCR. The amplification curves were observed only for CSFV, ASFV, and *E. rhusiopathiae*. Other viruses or bacterium, including PEDV, PDCoV, TGEV, SADS-CoV, FMDV-O, FMDV-A, PRRSV, PRV, PCV2, swine *Escherichia coli*, swine *Pasteurella multocida*, and nuclease-free water exhibited no fluorescent signals or amplification curves, indicating the assay’s high specificity (Figure 3).

### 3.4. Repeatability of the multiplex qRT-PCR

To assess the repeatability of the multiplex qRT-PCR, three different concentrations of standard plasmids (2.89 × 10^3^, 2.89 × 10^4^, and 2.89 × 10^5^ copies/μL) were utilized as templates for both intra- and inter-assay comparisons. The results reveal that the coefficients of variation (CVs) for both intra- and inter-assay of the Ct values were less than 5% (Table 3), suggesting the assay’s excellent repeatability.

**Table 3:** Reproducibility of this multiplex real-time PCR assay.

Target pathogens	The concentration of pUC57-CSFV-ASFV-ER (copies/μL)	Intra-assay (*n* = 9)		Inter-assay (*n* = 3 × 3)	
		CT (MEAN ± SD)	CV (%)	CT (MEAN ± SD)	CV (%)
CSFV	2.89 × 10^3^	32.66 ± 0.38	1.17	32.00 ± 0.48	1.49
	2.89 × 10^4^	28.87 ± 0.37	1.26	28.17 ± 0.65	2.29
	2.89 × 10^5^	25.43 ± 0.31	1.23	24.78 ± 0.62	2.49
ASFV	2.89 × 10^3^	31.0.31 ± 0.52	1.66	30.79 ± 0.82	2.66
	2.89 × 10^4^	27.78 ± 0.53	1.90	26.94 ± 0.42	1.56
	2.89 × 10^5^	24.03 ± 0.54	2.27	23.06 ± 0.87	3.79
*E. rhusiopathiae*	2.89 × 10^3^	31.77 ± 0.40	1.27	31.95 ± 0.72	2.26
	2.89 × 10^4^	27.33 ± 0.37	1.35	26.74 ± 0.91	3.41
	2.89 × 10^5^	23.50 ± 0.28	1.21	23.32 ± 0.80	3.42

### 3.5. Detection of the clinical samples

A total of 150 clinical samples were gathered from Guangdong Province, Southern China, during the period spanning from February 2021 to December 2022. These samples were subsequently analyzed using the established multiplex qRT-PCR method to verify its suitability. As a result, the positive rates of CSFV, ASFV, and *E. rhusiopathiae* were 1.33% (2/150), 0 (0/75), and 3.33% (5/150), respectively (Supplementary Table 1). And no co-infection among the three pathogens was found.

### 3.6. Comparison of multiplex qRT-PCR with single-plex commercial PCR kits

Upon comparing the results obtained from the multiplex qRT-PCR and single-plex commercial PCR, the analysis revealed a 100% concordance rate for both positive and negative samples (Table 4). Additionally, the diagnostic sensitivity and specificity were both 100%, indicating the high clinical value of the assay.

**Table 4:** Comparison of multiplex qRT-PCR with single-plex commercial PCR kits.

Target pathogens	Multiplex qRT-PCR		Single-plex commercial PCR kits		Coincidence rate
	Positive	Negative	Positive	Negative	
CSFV	2	20	2	20	100%
ASFV	8	20	8	20	100%
*E. rhusiopathiae*	5	20	5	20	100%

## 4. Discussion

CSFV, ASFV, and *E. rhusiopathiae* are significant pathogens severely damaging the global swine industry. In certain pig herds, there may be cases of co-infection between CSFV and ASFV, resulting in clinical manifestations and pathological changes that are difficult to differentiate in the field. Similar observations also exist between CSFV and *E. rhusiopathiae* (18, 19). The clinical symptoms and pathological changes exhibited by these pathogens can be similar, posing a challenge in accurately identifying the causative agent just through clinical indicators. Hence, it is essential to differentially detect these three pathogens through laboratory procedures to diagnose these diseases accurately. Multiplex qPCR is one of the best options among the different methods for diagnosing because it can rapidly, precisely, sensitively, and accurately identify several pathogenic nucleic acids in a single reaction. It is possible to achieve simultaneous rapid detection of multiple pathogens, which is significant for rapid diagnosis, prevention, and epidemiological study of mixed infectious diseases. It is expected to be more useful in some sudden outbreak areas (20). In this study, we used three sets of specific primers and corresponding probes to develop a multiplex TaqMan probe-based qRT-PCR system that can detect CSFV, ASFV, and *E. rhusiopathiae* simultaneously and differentially. With a detection limit of 289 copies per reaction, The assay demonstrated the ability to precisely detect CSFV, ASFV, and *E. rhusiopathiae*, while exhibiting no cross-reactivity with other porcine pathogens. Moreover, the intra- and inter-assay CVs were all less than 3.79%. Thus demonstrating a high level of specificity, sensitivity, and repeatability. Furthermore, the overall concordance rate between the multiplex qRT-PCR and single-plex commercial PCR kits was 100%. Lastly, the positive rates of CSFV, ASFV, and *E. rhusiopathiae* in 150 clinical samples were 1.33%, 0, and 3.33%, respectively, indicating that CSFV and *E. rhusiopathiae* were still sporadically prevalent in pig herds in southern China. Even without co-infection among the three pathogens found, significant efforts are necessary to prevent and control these pathogens.

In summary, using the genomic sequences of CSFV, ASFV, and *E. rhusiopathiae*, specific primers and probes were designed. After optimizing the reaction conditions, a TaqMan-probe-based multiplex qRT-PCR capable of simultaneous and differential detection of CSFV, ASFV, and *E. rhusiopathiae* was developed with high specificity, sensitivity, repeatability, and clinical value.

## Data availability statement

The original contributions presented in the study are included in the article/Supplementary material, further inquiries can be directed to the corresponding authors.

## Author contributions

LZ, X-HW, and QZ designed the primers and probes, optimized the reaction conditions, and wrote the manuscript. C-LJ, X-RZ, and S-JL tested the diagnostic method. D-HL and QZ designed the experiments and revised the manuscript. All authors contributed to the article and approved the submitted version.

## Funding

This study was supported by the following grants: the Project of President Funding of Guangdong Academy of Agricultural Sciences (202024), Guangzhou Basic and Applied Basic Research Foundation (202201011182), the Key-Area Research and Development Program of Guangdong Province (grant number 2019B020211005), and the grants from the Department of Agriculture and Rural Affairs of Guangdong Province (2022KJ119 and 2023KJ119).

## Conflict of interest

The authors declare that the research was conducted in the absence of any commercial or financial relationships that could be construed as a potential conflict of interest.

## Publisher’s note

All claims expressed in this article are solely those of the authors and do not necessarily represent those of their affiliated organizations, or those of the publisher, the editors and the reviewers. Any product that may be evaluated in this article, or claim that may be made by its manufacturer, is not guaranteed or endorsed by the publisher.
