# Supplementary material for: Development of a multiplex qRT-PCR assay for detection of classical swine fever virus, African swine fever virus, and Erysipelothrix rhusiopathiae
Source: Front Vet Sci. 2023 May 25;10:1183360. doi: 10.3389/fvets.2023.1183360 (PMC10248016; doi:10.3389/fvets.2023.1183360)
Supplement: Supplementary file 1 [file Table_1.DOCX]

**Supplementary Table 1** Detection of the clinical samples

| Sample ID | Date | Sample type | multiplex qRT-PCR | | | | | |
| --- | --- | --- | --- | --- | --- | --- | --- | --- |
|  |  |  | CSFV | | ASFV | | *E. rhusiopathiae* | |
|  |  |  | Ct value | Result | Ct value | Result | Ct value | Result |
| No.1 | Feb,2021 | Blood | NT | － | NT | － | NT | － |
| No.2 | Feb,2021 | Blood | NT | － | NT | － | NT | － |
| No.3 | Feb,2021 | Blood | NT | － | NT | － | NT | － |
| No.4 | Feb,2021 | Blood | NT | － | NT | － | NT | － |
| No.5 | Feb,2021 | Blood | NT | － | NT | － | NT | － |
| No.6 | Feb,2021 | Blood | NT | － | NT | － | NT | － |
| No.7 | Feb,2021 | Blood | NT | － | NT | － | NT | － |
| No.8 | Feb,2021 | Blood | NT | － | NT | － | NT | － |
| No.9 | Feb,2021 | Blood | NT | － | NT | － | NT | － |
| No.10 | Feb,2021 | Blood | NT | － | NT | － | NT | － |
| No.11 | Feb,2021 | Blood | NT | － | NT | － | NT | － |
| No.12 | Feb,2021 | Blood | NT | － | NT | － | NT | － |
| No.13 | Feb,2021 | Blood | NT | － | NT | － | NT | － |
| No.14 | Feb,2021 | Blood | NT | － | NT | － | NT | － |
| No.15 | Feb,2021 | Blood | NT | － | NT | － | NT | － |
| No.16 | Feb,2021 | Blood | NT | － | NT | － | NT | － |
| No.17 | Feb,2021 | Blood | NT | － | NT | － | NT | － |
| No.18 | Feb,2021 | Blood | NT | － | NT | － | NT | － |
| No.19 | Feb,2021 | Blood | NT | － | NT | － | NT | － |
| No.20 | Feb,2021 | Blood | NT | － | NT | － | NT | － |
| No.21 | Feb,2021 | Blood | NT | － | NT | － | NT | － |
| No.22 | Feb,2021 | Blood | NT | － | NT | － | NT | － |
| No.23 | Feb,2021 | Blood | NT | － | NT | － | NT | － |
| No.24 | Feb,2021 | Blood | NT | － | NT | － | NT | － |
| No.25 | Feb,2021 | Blood | NT | － | NT | － | NT | － |
| No.26 | Feb,2021 | Blood | NT | － | NT | － | NT | － |
| No.27 | Feb,2021 | Blood | NT | － | NT | － | NT | － |
| No.28 | Mar,2021 | Lymph node | NT | － | NT | － | NT | － |
| No.29 | Mar,2021 | Lymph node | NT | － | NT | － | NT | － |
| No.30 | Mar,2021 | Lymph node | NT | － | NT | － | NT | － |
| No.31 | Mar,2021 | Lymph node | NT | － | NT | － | NT | － |
| No.32 | Mar,2021 | Lymph node | NT | － | NT | － | NT | － |
| No.33 | Mar,2021 | Lymph node | NT | － | NT | － | NT | － |
| No.34 | Mar,2021 | Lymph node | NT | － | NT | － | NT | － |
| No.35 | Mar,2021 | Lymph node | NT | － | NT | － | NT | － |
| No.36 | Mar,2021 | Lymph node | NT | － | NT | － | NT | － |
| No.37 | Mar,2021 | Lymph node | NT | － | NT | － | NT | － |
| No.38 | Mar,2021 | Lymph node | NT | － | NT | － | NT | － |
| No.39 | Mar,2021 | Lymph node | NT | － | NT | － | NT | － |
| No.40 | Mar,2021 | Lymph node | NT | － | NT | － | NT | － |
| No.41 | Mar,2021 | Blood | NT | － | NT | － | NT | － |
| No.42 | Mar,2021 | Blood | NT | － | NT | － | NT | － |
| No.43 | Mar,2021 | Blood | NT | － | NT | － | NT | － |
| No.44 | Mar,2021 | Blood | NT | － | NT | － | NT | － |
| No.45 | Mar,2021 | Blood | NT | － | NT | － | NT | － |
| No.46 | Mar,2021 | Blood | NT | － | NT | － | NT | － |
| No.47 | Mar,2021 | Blood | NT | － | NT | － | NT | － |
| No.48 | Mar,2021 | Blood | NT | － | NT | － | NT | － |
| No.49 | Mar,2021 | Blood | NT | － | NT | － | NT | － |
| No.50 | Mar,2021 | Blood | NT | － | NT | － | NT | － |
| No.51 | Aug,2021 | Lymph node | NT | － | NT | － | NT | － |
| No.52 | Aug,2021 | Lymph node | NT | － | NT | － | NT | － |
| No.53 | Aug,2021 | Lymph node | NT | － | NT | － | NT | － |
| No.54 | Aug,2021 | Lymph node | NT | － | NT | － | NT | － |
| No.55 | Aug,2021 | Lymph node | NT | － | NT | － | NT | － |
| No.56 | Aug,2021 | Blood | NT | － | NT | － | NT | － |
| No.57 | Aug,2021 | Blood | NT | － | NT | － | NT | － |
| No.58 | Aug,2021 | Blood | NT | － | NT | － | NT | － |
| No.59 | Aug,2021 | Blood | 16.19 | + | NT | － | NT | － |
| No.60 | Aug,2021 | Blood | NT | － | NT | － | NT | － |
| No.61 | Aug,2021 | Blood | NT | － | NT | － | NT | － |
| No.62 | Aug,2021 | Blood | NT | － | NT | － | NT | － |
| No.63 | Aug,2021 | Blood | NT | － | NT | － | NT | － |
| No.64 | Nov,2021 | Blood | NT | － | NT | － | NT | － |
| No.65 | Nov,2021 | Blood | NT | － | NT | － | NT | － |
| No.66 | Nov,2021 | Blood | NT | － | NT | － | NT | － |
| No.67 | Nov,2021 | Blood | NT | － | NT | － | NT | － |
| No.68 | Nov,2021 | Blood | NT | － | NT | － | NT | － |
| No.69 | Nov,2021 | Blood | NT | － | NT | － | NT | － |
| No.70 | Nov,2021 | Blood | NT | － | NT | － | NT | － |
| No.71 | Nov,2021 | Blood | NT | － | NT | － | NT | － |
| No.72 | Nov,2021 | Blood | NT | － | NT | － | NT | － |
| No.73 | Mar,2022 | Kidney | NT | － | NT | － | 31.80 | + |
| No.74 | Mar,2022 | Kidney | NT | － | NT | － | 34.29 | + |
| No.75 | Mar,2022 | Kidney | NT | － | NT | － | NT | － |
| No.76 | May,2022 | Blood | NT | － | NT | － | NT | － |
| No.77 | May,2022 | Blood | NT | － | NT | － | NT | － |
| No.78 | May,2022 | Blood | NT | － | NT | － | NT | － |
| No.79 | May,2022 | Blood | NT | － | NT | － | NT | － |
| No.80 | May,2022 | Blood | NT | － | NT | － | NT | － |
| No.81 | May,2022 | Blood | NT | － | NT | － | 23.93 | + |
| No.82 | May,2022 | Blood | NT | － | NT | － | NT | － |
| No.83 | May,2022 | Blood | NT | － | NT | － | NT | － |
| No.84 | May,2022 | Blood | NT | － | NT | － | NT | － |
| No.85 | May,2022 | Blood | NT | － | NT | － | NT | － |
| No.86 | May,2022 | Blood | NT | － | NT | － | NT | － |
| No.87 | May,2022 | Blood | NT | － | NT | － | NT | － |
| No.88 | May,2022 | Blood | NT | － | NT | － | NT | － |
| No.89 | May,2022 | Blood | NT | － | NT | － | NT | － |
| No.90 | May,2022 | Blood | NT | － | NT | － | NT | － |
| No.91 | May,2022 | Blood | NT | － | NT | － | NT | － |
| No.92 | May,2022 | Blood | NT | － | NT | － | NT | － |
| No.93 | May,2022 | Blood | NT | － | NT | － | NT | － |
| No.94 | May,2022 | Blood | NT | － | NT | － | NT | － |
| No.95 | May,2022 | Blood | NT | － | NT | － | NT | － |
| No.96 | May,2022 | Blood | NT | － | NT | － | NT | － |
| No.97 | May,2022 | Blood | NT | － | NT | － | NT | － |
| No.98 | May,2022 | Blood | NT | － | NT | － | NT | － |
| No.99 | May,2022 | Blood | NT | － | NT | － | NT | － |
| No.100 | May,2022 | Blood | NT | － | NT | － | NT | － |
| No.101 | Aug,2022 | Lymph node | NT | － | NT | － | NT | － |
| No.102 | Aug,2022 | Lymph node | NT | － | NT | － | 27.15 | + |
| No.103 | Aug,2022 | Lymph node | NT | － | NT | － | NT | － |
| No.104 | Aug,2022 | Lymph node | NT | － | NT | － | 26.91 | + |
| No.105 | Aug,2022 | Lymph node | NT | － | NT | － | NT | － |
| No.106 | Aug,2022 | Lymph node | NT | － | NT | － | NT | － |
| No.107 | Aug,2022 | Lymph node | NT | － | NT | － | NT | － |
| No.108 | Aug,2022 | Lymph node | NT | － | NT | － | NT | － |
| No.109 | Aug,2022 | Lymph node | NT | － | NT | － | NT | － |
| No.110 | Aug,2022 | Lymph node | NT | － | NT | － | NT | － |
| No.111 | Aug,2022 | Lymph node | NT | － | NT | － | NT | － |
| No.112 | Aug,2022 | Lymph node | NT | － | NT | － | NT | － |
| No.113 | Aug,2022 | Lymph node | NT | － | NT | － | NT | － |
| No.114 | Aug,2022 | Lymph node | NT | － | NT | － | NT | － |
| No.115 | Aug,2022 | Lymph node | NT | － | NT | － | NT | － |
| No.116 | Aug,2022 | Lymph node | NT | － | NT | － | NT | － |
| No.117 | Aug,2022 | Lymph node | NT | － | NT | － | NT | － |
| No.118 | Aug,2022 | Blood | NT | － | NT | － | NT | － |
| No.119 | Aug,2022 | Blood | NT | － | NT | － | NT | － |
| No.120 | Aug,2022 | Blood | NT | － | NT | － | NT | － |
| No.121 | Aug,2022 | Blood | NT | － | NT | － | NT | － |
| No.122 | Aug,2022 | Blood | NT | － | NT | － | NT | － |
| No.123 | Aug,2022 | Blood | NT | － | NT | － | NT | － |
| No.124 | Aug,2022 | Blood | NT | － | NT | － | NT | － |
| No.125 | Aug,2022 | Blood | NT | － | NT | － | NT | － |
| No.126 | Aug,2022 | Blood | NT | － | NT | － | NT | － |
| No.127 | Aug,2022 | Blood | 22.35 | + | NT | － | NT | － |
| No.128 | Aug,2022 | Blood | NT | － | NT | － | NT | － |
| No.129 | Aug,2022 | Blood | NT | － | NT | － | NT | － |
| No.130 | Aug,2022 | Blood | NT | － | NT | － | NT | － |
| No.131 | Dec,2022 | Blood | NT | － | NT | － | NT | － |
| No.132 | Dec,2022 | Blood | NT | － | NT | － | NT | － |
| No.133 | Dec,2022 | Blood | NT | － | NT | － | NT | － |
| No.134 | Dec,2022 | Blood | NT | － | NT | － | NT | － |
| No.135 | Dec,2022 | Blood | NT | － | NT | － | NT | － |
| No.136 | Dec,2022 | Blood | NT | － | NT | － | NT | － |
| No.137 | Dec,2022 | Blood | NT | － | NT | － | NT | － |
| No.138 | Dec,2022 | Blood | NT | － | NT | － | NT | － |
| No.139 | Dec,2022 | Blood | NT | － | NT | － | NT | － |
| No.140 | Dec,2022 | Blood | NT | － | NT | － | NT | － |
| No.141 | Dec,2022 | Blood | NT | － | NT | － | NT | － |
| No.142 | Dec,2022 | Blood | NT | － | NT | － | NT | － |
| No.143 | Dec,2022 | Blood | NT | － | NT | － | NT | － |
| No.144 | Dec,2022 | Blood | NT | － | NT | － | NT | － |
| No.145 | Dec,2022 | Blood | NT | － | NT | － | NT | － |
| No.146 | Dec,2022 | Kidney | NT | － | NT | － | NT | － |
| No.147 | Dec,2022 | Kidney | NT | － | NT | － | NT | － |
| No.148 | Dec,2022 | Kidney | NT | － | NT | － | NT | － |
| No.149 | Dec,2022 | Kidney | NT | － | NT | － | NT | － |
| No.150 | Dec,2022 | Kidney | NT | － | NT | － | NT | － |
